# Genome Mining and Evaluation of the Biocontrol Potential of *Pseudomonas fluorescens* BRZ63, a New Endophyte of Oilseed Rape (*Brassica napus* L.) against Fungal Pathogens

**DOI:** 10.3390/ijms21228740

**Published:** 2020-11-19

**Authors:** Daria Chlebek, Artur Pinski, Joanna Żur, Justyna Michalska, Katarzyna Hupert-Kocurek

**Affiliations:** Institute of Biology, Biotechnology and Environmental Protection, Faculty of Natural Sciences, University of Silesia in Katowice, Jagiellonska 28, 40-032 Katowice, Poland; apinski@us.edu.pl (A.P.); joanna.zur@us.edu.pl (J.Ż.); jmichalska@us.edu.pl (J.M.)

**Keywords:** biocontrol, endophytic bacteria, genome mining, oilseed rape, phytopathogens, plant growth promotion, *Pseudomonas fluorescens*

## Abstract

Endophytic bacteria hold tremendous potential for use as biocontrol agents. Our study aimed to investigate the biocontrol activity of *Pseudomonas fluorescens* BRZ63, a new endophyte of oilseed rape (*Brassica napus* L.) against *Rhizoctonia solani* W70, *Colletotrichum dematium* K, *Sclerotinia sclerotiorum* K2291, and *Fusarium avenaceum*. In addition, features crucial for biocontrol, plant growth promotion, and colonization were assessed and linked with the genome sequences. The in vitro tests showed that BRZ63 significantly inhibited the mycelium growth of all tested pathogens and stimulated germination and growth of oilseed rape seedlings treated with fungal pathogens. The BRZ63 strain can benefit plants by producing biosurfactants, siderophores, indole-3-acetic acid (IAA), 1-aminocyclopropane-1-carboxylate (ACC) deaminase, and ammonia as well as phosphate solubilization. The abilities of exopolysaccharide production, autoaggregation, and biofilm formation additionally underline its potential to plant colonization and hence biocontrol. The effective colonization properties of the BRZ63 strain were confirmed by microscopy observations of EGFP-expressing cells colonizing the root surface and epidermal cells of *Arabidopsis thaliana* Col-0. Genome mining identified many genes related to the biocontrol process, such as transporters, siderophores, and other secondary metabolites. All analyses revealed that the BRZ63 strain is an excellent endophytic candidate for biocontrol of various plant pathogens and plant growth promotion.

## 1. Introduction

In recent years, the yield and the quality of many crops and medicinal plants, as well as vegetables and fruits have decreased because of plant diseases caused by soil-borne pathogens, including fungi. Fungal pathogens are responsible for a range of serious plant diseases, such as *Fusarium* wilt, highly destructive to tomatoes [1]; phytophthora blight, the most destructive disease caused by the infection of oomycete pathogen *Phytophthora capsici* [2]; *Rhizoctonia* diseases of many crops, cotton, and lettuce [3]; or *Sclerotinia* stem rot (SSR), reducing the yield of rapeseed [4], a major source of vegetable oil for human consumption and biodiesel production [5]. Among different methods used for controlling soil-borne pathogens, chemical control based on synthetic products dominates. However, the excessive use of such compounds results in the reduction of agricultural product quality and, with time, in pathogen resistance to the applied chemicals. In a long-term perspective, this leads to the destruction of ecosystems and damage to human health [6,7]. Thus, finding eco-friendly methods of plant disease control is of paramount importance [8]. Biocontrol offers a more sustainable alternative to chemical pesticides [9] as plant pathogens are controlled through natural antagonists [10]. Recently, special attention has focused on endophytic bacteria, symbionts residing within the plant tissues for the majority of their life cycle that not only remain asymptomatic but also have beneficial effects on their host [11]. Given the intimate contact with plants, these microorganisms hold tremendous potential for use as biocontrol agents [12].

Efficient biocontrol of phytopathogens is the result of several mechanisms, which include antibiosis, competition for nutrients and niches, production of cell wall-degrading enzymes, synthesis of biosurfactants and volatile organic and inorganic compounds, and/or induced systemic resistance [13]. Additionally, colonization of plants by endophytes induces several cell wall modifications, such as deposition of callose, pectin, cellulose, and phenolic compounds, leading to the formation of a structural barrier at the site of the potential attack by phytopathogens [14]. Endophytic colonization can also result in increased expression of extensins, which plays an important role in plant defense by strengthening the cell wall. It is worth noting that changes in the cell wall structural proteins and enzymes seems to be strain dependent [15]. In biocontrol activity, multiple mechanisms and their synergistic effects might be involved [16].

Although several bacteria species are known for their antimicrobial activity, *Pseudomonas* spp. are one of the most important in agriculture and have long been known to be beneficial to plants because of their potential as biological control agents [17,18] and plant growth-promoting effects [19]. Furthermore, these microorganisms are involved in phytoremediation processes because they play a vital role in degradation of petroleum hydrocarbons as well as other mono- and polycyclic aromatic hydrocarbons, which is enhanced by their ability to produce biosurfactants (e.g., rhamnolipid) [20]. Until now, over 200 species of the *Pseudomonas* genus have been described [21] and a number of them have been classified as *Pseudomonas fluorescens*.

In this work, a new *Pseudomonas fluorescens* BRZ63 strain, isolated from roots of the oilseed rape (*Brassica napus* L.), was investigated to identify both its endophytic ability and biocontrol traits. We combined traditional microbiological methods with in silico genomic analysis, aiming to reveal the genetic background of these properties. The main objectives of the study were: (1) determination of the antifungal activity of the tested strain against fungal phytopathogens, (2) evaluation of the endophytic behavior and biocontrol features of the strain by in vitro bioassays, (3) determination of the ability of the strain to colonize the root surface and internal tissues of plants, (4) in vitro evaluation of the effects of the BRZ63 strain on oilseed rape fitness and protection against fungal disease, and (5) identification of genes crucial for colonization of plants, biocontrol activity, and plant growth promotion in the genome of the BRZ63 strain.

## 2. Results

### 2.1. Antagonistic Activity of P. fluorescens BRZ63 against Fungal Plant Pathogens

**Figure 1:**
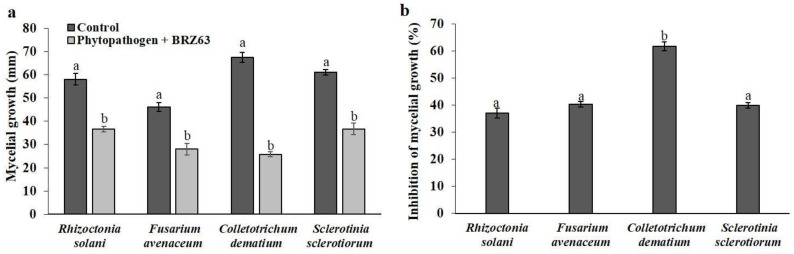
Effect of *P. fluorescens* BRZ63 on mycelial growth (**a**) and percentage (%) of inhibition of fungal phytopathogens’ mycelial growth by the BRZ63 strain (**b**). Each column represents the mean ± standard deviation of three independent replications for each treatment. a,b−indicate significant differences (*p* < 0.05) according to the honest significant difference (HSD) test.

The antagonism test results showed that the BRZ63 strain reduced and inhibited the mycelium growth of all plant pathogens used in this study (Figure 1a, Figure S1). The growth of selected phytopathogens was reduced by the BRZ63 strain by different extents and the inhibition of growth was significant in comparison with the negative controls. The strongest inhibitory effect was observed against *C. dematium* K, where the mycelium expansion was inhibited by over 61.8 ± 1.64%. The BRZ63 strain inhibited the growth of *S. sclerotiorum* K2291 by 39.9 ± 1.08%, followed by *F. avenaceum* (40.33 ± 1.04%) and *R. solani* W70 (37.02 ± 2.85%) (Figure 1b).

### 2.2. In Vitro Assessment of Colonization Properties of the BRZ63 Strain

**Figure 2:**
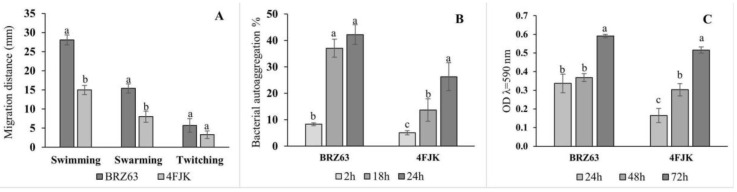
Motility (**A**), autoaggregation (**B**), and biofilm formation ability (**C**) of *P. fluorescens* BRZ63. The *Enterobacter asburiae* 4FJK strain was used as a control strain. The autoaggregation ability of the tested strains is expressed as a percentage of aggregated cells after 2, 18, and 24 h of incubation. The biofilm formation was assessed over a period of 72 h by using the crystal violet (CV) method. Each column represents the mean ± standard deviations of three independent replications for each treatment. a,b,c−indicate statistically significant differences (*p* < 0.05) according to the HSD test.

Motility assays revealed the ability of the studied strain to spread on 0.3% (swimming), 0.5% (swarming), and 1% (twitching) agar plates. The distance was measured from the center of the original inoculation spot to the leading edge of bacterial growth. The results summarized in Figure 2A show that the BRZ63 strain exhibited a higher motility than the control endophytic strain. When it was cultivated on the agar plates, the distance of migration (28.1 ± 1.3 mm) was bigger than that on the swarm agar plates (15.4 ± 1.2 mm), while onto twitch agar, the BRZ63 strain remained near the point of inoculation (5.7 ± 0.8 mm). The BRZ63 strain formed reddish black colonies with a crystalline consistency on the Congo Red Agar (CRA) medium, which confirmed its ability of exopolysaccharide (EPS) production (Table 1). Another important feature of the strain is its autoaggregation and biofilm formation ability. The results obtained in this study showed that the BRZ63 strain was self-aggregating, with the autoaggregation index increasing with the time of incubation up to 42.21 ± 3.7% after 24 h. The reference endophytic strain 4FJK showed a lower ability to autoaggregate (26 ± 4.30%) (Figure 2B). The ability of the BRZ63 strain to develop a biofilm on polystyrene microtiter dishes was assessed using the crystal violet (CV) method. The obtained results showed that the tested strain formed biofilm after 24 h of incubation (Figure 2C), and based on the classification of Stepanović et al. [22], it was classified as a moderate biofilm producer. In addition to the traits described above, the BRZ63 strain exhibited oxidase and catalase activity (Table 1).

### 2.3. Inoculation of Arabidopsis Thaliana Col-0 with the EGFP-Labeled Strain

**Figure 3:**
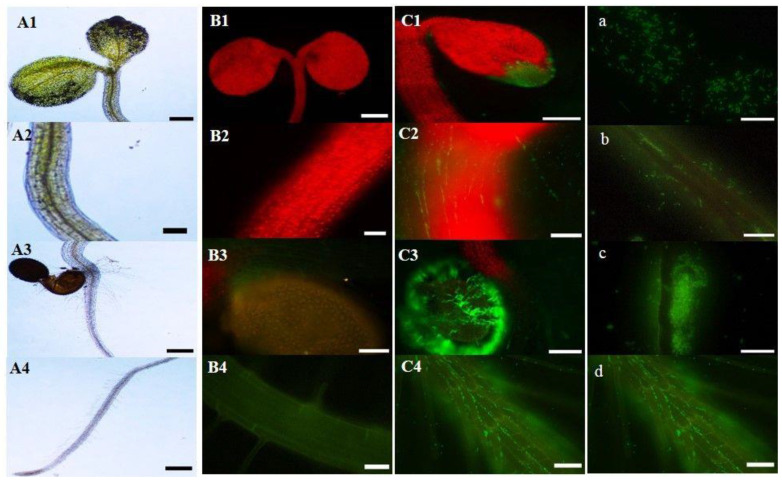
The colonization of *Arabidopsis thaliana* Col-0 and stages of the plant roots colonization with the EGFP-labeled BRZ63 strain. Uninoculated plant observed under a light microscope (**A1**–**A4**) (scale bar: A1 = 200 µm, A2 = 100 µm, A3 = 200 µm, A4 = 200 µm). Uninoculated plant (**B1**–**B4**) (scale bar: B1 = 100 µm, B2 = 100 µm, B3 = 100 µm, B4 = 40 µm, and plant after inoculation with EGFP-labeled BRZ63 strain (**C1**–**C4**) (scale bar: C1 = 200 µm, C2 = 50 µm, C3 = 200 µm, C4 = 20 µm observed with a Nikon ECLIPSE-Ni-U stereo microscope equipped with epifluorescence detection. EGFP-labeled cells of BRZ63 (scale bar = 10 µm) (**a**), adhesion of bacteria to the root surface and the root hairs (scale bar = 20 µm) (**b**), biofilm formation (scale bar = 20 µm) (**c**), colonization of epidermal cells (scale bar = 20 µm) (**d**). All pictures were processed using ImageJ software.

Fluorescently labeled bacteria are handy tools to study endophyte–plant interaction. In this study, pMP4566 plasmid with the cloned *egfp* gene [23] was successfully transformed into the BRZ63 strain (Figure 3a). To verify that the strain could colonize plant tissues and thrive within them, surface-sterilized seeds of *Arabidopsis thaliana* Col-0 were inoculated with the EGFP-labeled strain and grown on water agar plates. After 7 days, 6 seedlings were selected and the fluorescence of the labeled BRZ63 strain population in planta was observed (Figure 3(C1–C4). As it is shown in Figure 3b–d, EGFP-expressing cells adhered and formed biofilm on the root surface, and colonized epidermal cells of the model plant.

### 2.4. Effects of the BRZ63 Strain on Plant Development and Disease Protection of Oilseed Rape

**Figure 4:**
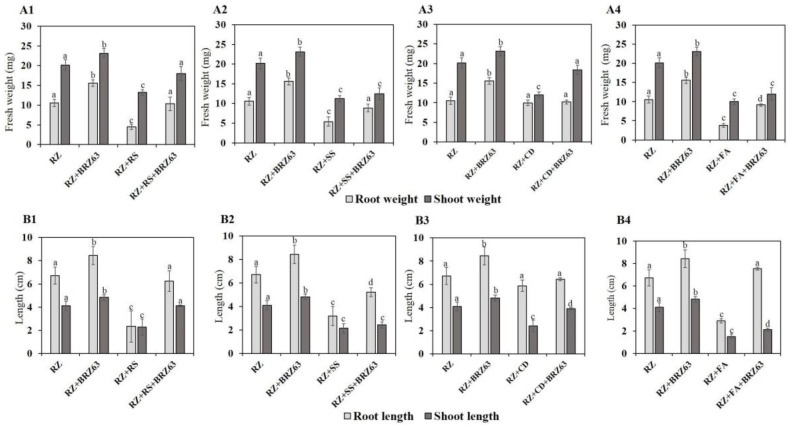
Effects of the BRZ63 strain on plant development and disease protection of oilseed rape. The root and shoot weights of germinating seeds after different treatments (**A1**–**A4**). The root and shoot lengths of germinating seeds after different treatments (**B1**–**B4**). The different letters (a–d) represent significant difference (*p* < 0.05) in root/shoot lengths and root/shoot weights of germinating seeds. The treatments are described in Section 4.8.

**Figure 5:**
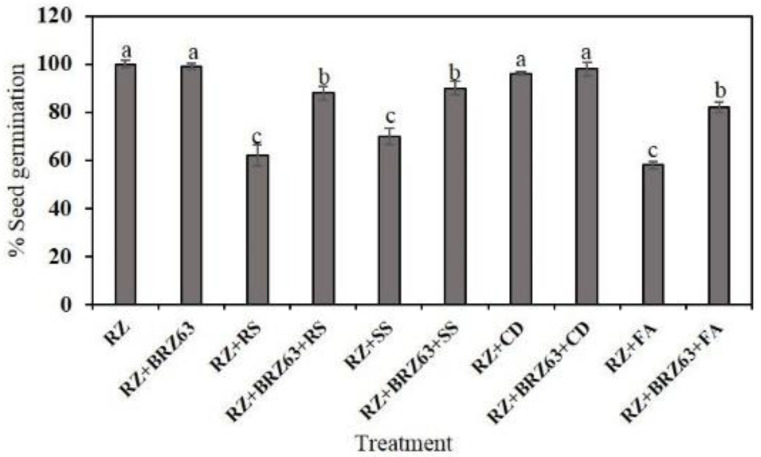
Germination efficiency of oilseed rape seeds after different treatments. The different letters (a–c) above the bar represent significant differences (*p* < 0.05) in the germination percentage. The treatments are described in Section 4.8.

The BRZ63 strain stimulated the growth of oilseed rape seedlings both not exposed to and treated with fungal pathogens. Significant differences were observed between plants inoculated only with pathogen and those co-inoculated with the fungal strain and the examined BRZ63 strain. Preinoculation of seeds with the BRZ63 strain increased the weight and root length of seedlings exposed to *R. solani* W70, *S. sclerotiorum* K2291, and *F. avenaceum* (Figure 4). Importantly, the BRZ63 strain increased the weight and shoot length of seedlings grown in the presence of the *C. dematium* K (Figure 4). Additionally, except for the *C. dematium* treatment, the oilseed rape seeds co-inoculated with fungi and the BRZ63 strain showed higher germination efficiency than uninoculated seeds exposed to mycelial mass of fungal pathogens. The germination percentage for each treatment is represented in Figure 5. Moreover, the general disease symptoms: yellow chlorosis in leaves, root brown lesions, and necrosis, were significantly reduced in plants co-inoculated with fungal pathogens and the BRZ63 strain (Figure S2). These results clearly demonstrate plant-protective effects of the BRZ63 strains against selected pathogens.

### 2.5. In Vitro Screening for PGP and Biocontrol Traits of the BRZ63 Strain

The results of the biochemical assays revealed that *P. fluorescens* BRZ63 produced a high level of indole-3-acetic acid (IAA) (59.62 ± 1.11 μg/mL) in the presence of tryptophan (Table 1). It was also able to produce salicylic acid (SA) in the concentration of 17.83 ± 0.95 μg/mL in succinate medium (Table 1). Another important mechanism that promotes plant growth is the ability of endophytes to produce ACC deaminase, reducing the level of ethylene in the plant. The result obtained in this study showed that the BRZ63 strain was able to grow on Dworkin and Foster (DF) minimal salt medium supplemented with 3 mM ACC as a nitrogen source, implying ACC deaminase activity (Table 1). The BRZ63 strain was found to have a phosphate solubilization index (PSI) of 7.67 ± 0.33. The significantly lower PSI was exhibited by the control strain 4FJK (1.76 ± 0.19) (Table 2). The BRZ63 strain was also positive in the reaction with Nessler’s reagent. A dark brown color was observed, indicating ammonia production. The chrome azurol S (CAS) agar plates were used to observe the siderophores production by the strain. The orange halo around strain colonies indicated the ability of the BRZ63 strain to produce siderophores (Table 1). The BRZ63 strain was also positive for endoglucanase and chitinase production (Table 1). Biosurfactant production is another metabolic capability of the BRZ63 strain, which can be harnessed for biocontrol. The positive reaction for methylene blue agar tests suggests the rhamnolipid nature of the biosurfactant produced by the BRZ63 strain (Table 3). The tested strain demonstrated high emulsification activity (78.79 ± 2.62% and 32.5 ± 2.5% with cyclohexane and diesel oil, respectively). Furthermore, the produced biosurfactants showed great potential for lowering the surface tension (40.30 ± 3.50 mm). The results of the qualitative (CTAB/methylene blue agar test) and semi-quantitative (emulsification index; lowering the surface tension) parameters obtained by the BRZ63 strain were similar to those obtained for the reference P-1 strain (Table 3).

### 2.6. General Features of P. fluorescens BRZ63 Genome and Phylogenetic Analysis

Following the determination of antagonistic activity and biochemical characterization of *P. fluorescens* BRZ63, whole-genome sequence analysis was performed. The general genome features of the BRZ63 strain are summarized in Table 4. The assembly of the genome sequence resulted in 363 contigs with an estimated genome size of 6,335,040 bp, and an average of G+C content of 64%. In total, 6120 genes, of which 5915 were annotated as protein-encoding sequences (CDSs), 77 RNAs and 128 pseudogenes were predicted, which was similar to the results reported for other endophytic *P. fluorescens* strains [18,24,25]. Of the total CDSs, 5949 genes were classified into clusters of orthologous group (COG) families comprised of 22 categories (Table S1). It revealed three main functional gene classes: amino acid transport and metabolism (E category), inorganic ion transport (P category), and carbohydrate transport and metabolism (G category). Especially, the number of genes participating in transport and metabolism of amino acids and carbohydrates indicates the inherent capacity of the BRZ63 strain to survive and compete with other microorganisms in the rhizosphere/endosphere. In addition, the number of genes participating in cell motility (C category) identified emphasizes the ability of the BRZ63 strain to effectively colonize plants [26] (Table S1). A large number of predicted genes were assigned as encoding proteins involved in secondary metabolites biosynthesis, transport, and catabolism (Q category). The antiSMASH analysis also disclosed secondary metabolite clusters in the genome of the BRZ63 strain (Table S2).

**Figure 6:**
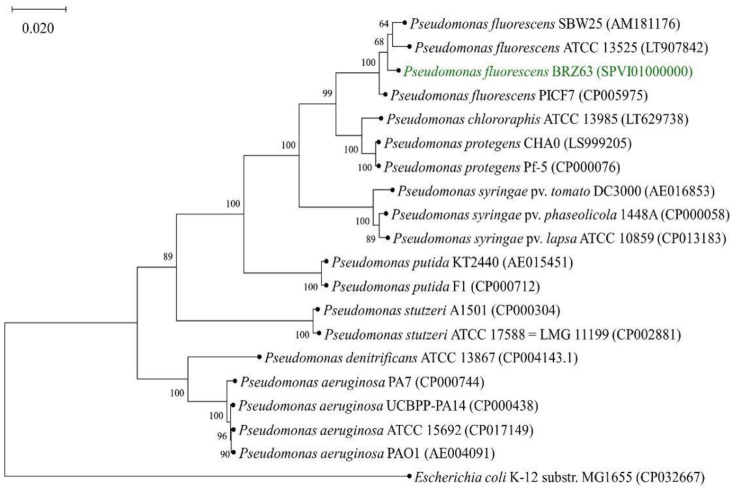
Phylogenetic tree of *Pseudomonas fluorescens* BRZ63 (marked in green) based on alignment of the core proteome of 20 strains with *Escherichia coli* K-12 substr. MG1655 as an outgroup. Branching percentage values were determined with the use of the 1000 bootstraps resampling test. The accession numbers for genomes are shown in brackets. The tree was constructed using the maximum likelihood method. The scale bar represents 1% nucleotide sequence divergence.

The phylogenetic tree including *P. fluorescens* BRZ63 based on the alignment of the core proteome of 20 strains is illustrated in Figure 6. In the phylogenetic group of genus *Pseudomonas*, BRZ63 grouped closely with *Pseudomonas fluorescens* SBW25 and ATCC 13525.

### 2.7. The Colonization, Biocontrol, and PGP Features in the BRZ63 Genome

The KEGG annotation and the functional annotation of proteins based on the eggNOG protein database revealed genes related to plant–bacteria interaction, biocontrol, and PGP traits (Table S3). The BRZ63 strain carries clusters containing *flg*, *fil*, and *flh* genes encoding the machinery for flagella biosynthesis and motility. The genome annotations revealed also *cheA*, *cheB*, *cheW*, and *mpc* genes encoding proteins of the most widespread bacterial chemotaxis signaling pathway as well as genes for additional chemotaxis proteins: CheB, CheR, CheZ, and CheV. Furthermore, in the BRZ63 genome, genes required for type IV pili biosynthesis were detected. Among the features essential for bacterial adherence to surfaces during the initial stage of biofilm formation, the presence of 12 *alg* genes encoding proteins involved in alginate biosynthesis and key genes of lipopolysaccharide (LPS) biosynthesis, including operons *lpx*, was admitted. In the genome of BRZ63, we identified 134 putative genes encoding CAZy distributed unevenly among the six CAZy families (Table S4). Within the class of glycoside hydrolases (GH) and carbohydrate esterases (CE), CAZymes with the potential to degrade many cell wall polymers, including beta-1,3-glucan, cellulose, hemicellulose, pectin, peptidoglycanes, polysaccharides, and chitooligosaccharides, were found (Table S5). An advantageous feature of the strain is the presence of genes encoding various lytic enzymes, such as beta-glucosidase (*bglX*) and N-acetylglucosamine-6-phosphate deacetylase (*nagAl*). Moreover, analysis of the BRZ63 genome revealed the presence of several genes encoding catalases, glutathione S-transferase, glutathione reductase, superoxide dismutase [Fe], and superoxide dismutase [Mn/Fe], which protect plant defense mechanisms.

As potential biocontrol strain, we sought genes involved in the production of antimicrobial molecules. In the genome of the BRZ63 strain, genes crucial for phenazine biosynthesis (*phzF* encoding trans-2,3-dihydro-3-hydroxyanthranilate isomerase and *trdG* encoding anthranilate synthase component), *ubiC* gene encoding chorismate pyruvate-lyase involved in the biosynthesis of 4-hydroxybenzoate, as well as genes encoding pyocin-S2 (*pys2*) and oligopeptidase A (*prlC*), were identified. Moreover, the BRZ63 strain carried genes involved in the synthesis of acetoin and butanediol that act as growth-promoting factors and increase plant resistance against pathogens.

Analysis of the BRZ63 genome also revealed the presence of a number of genes involved in siderophore production and transport along with genes encoding bacterioferritin, enterobactin, and pyoverdine. Furthermore, we found genes for TonB-dependent receptors (TBDRs), indicating that the BRZ63 strain may be highly effective in the competitive acquisition of iron. In addition to these features, the strain also contains tryptophan biosynthesis genes (*trpABCDE*) and genes encoding oxidoreductase, aldoxime dehydratase, and nitrilase. In addition, a set of putative proteins involved in nitrogen fixation (*iscU*) and phosphate metabolism and transport (operon *pstABCS* encoding permease proteins (PstA and PstC), the phosphate import protein (PstB), and the phosphate binding protein (PstS)) was identified. Genome examination also showed several genes coding for the Pho system: *phoQ*, *phoP*, and *phoU* together with the gene responsible for glutamate dehydrogenase (GDH) synthesis (*gcd*) and the *pqq* gene cluster (*pqqABCDE*) necessary for the biosynthesis of PQQ cofactor.

## 3. Discussion

In this study, we characterized *P. fluorescens* BRZ63, a new endophyte of oilseed rape (*Brassica napus* L.), and demonstrated its phenotypic functionality related to the inhibition of the growth of fungal pathogens, biocontrol, and plant growth promotion. Moreover, the results of phenotypic analysis were linked with the genome sequences analyses for the understanding of genome–phenotype interplays. Many strains of *P. fluorescens* have been shown to be potential biocontrol agents suppressing plant pathogens. However, they were isolated mainly from the rhizosphere or the bulk soil [27,28,29,30,31]. To our knowledge, little is known about the biocontrol activity of *P. fluorescens* strains originally isolated from the internal tissues of rape. It is worth emphasizing that, in contrast with soil and rhizosphere bacteria, endophytic microorganisms could offer systemic tolerance against many plant pathogens as they colonize the entire plant tissues [32]. Furthermore, the plant growth-promoting potential of endophytes is higher than that of soil or rhizosphere microorganisms as they closely interact with their host plants [33]. Endophytes can also colonize seeds and support the germination process [34].

To potentially use *P. fluorescens* BRZ63 as a biocontrol agent, we checked if it could act against some ubiquitous soil-borne plant pathogenic fungi responsible for significant yield losses of crops, including rape [35,36]. In vitro tests demonstrated that the BRZ63 strain, which is closely related to *P. fluorescens* PICF7, the effective biocontrol agent against *Verticillium dahlia* [18], had a positive antagonistic effect on mycelial growth of *R. solani*, *S. sclerotiorum*, *F. avenaceum*, and *Colletotrichum* spp. This emphasizes the potential of the strain to protect plants from various fungal pathogens’ invasion and suggests involvement of various mechanisms in this process. To verify these assumptions, we explored the biocontrol efficacy of *P. fluorescens* BRZ63 against fungal pathogens on germinating oilseed rape seeds. The BRZ63 strain was found to confer protection to oilseed rape against all fungi tested in this study despite the different infection strategies used by the pathogens. In case of *R. solani* and *F. avenaceum*, the common root-infecting pathogens causing severe yield reductions in canola [37,38], preinoculation of seeds provided promotion of the length and fresh weights of roots while in plants treated with *C. dematium*, the fungus infecting leaves, flowers, and stems of the host plants [39], the BRZ63 strain caused a limitation of the disease symptoms manifested by lowering the length and fresh weight of the shoots. Among related *Pseudomonas* strains, *P. protegens* Pf-5 suppressed the growth of *R. solani*, *Sclerotinia homoeocarpa*, and *Fusarium oxysporum* [40] while *P. fluorescens* SBW25 and *P. protegens* CHAO were able to protect peas from seedling damping-off caused by the oomycete *Pythium ultimatum* [41].

The BRZ63 strain showed a wide spectrum of crucial features for successful colonization of plants. First of all, it exhibited a swimming, swarming, and twitching motility phenotype. The results were consistent with identification of flagellar genes and genes required for chemotaxis, adhesion, and type IV pili biosynthesis. The motility of bacteria, dependent on the presence of flagella or pili, is one of the crucial factors that promotes attachment to different surfaces and biofilm formation [42,43]. The involvement of the flagellar biosynthesis genes in plant colonization was shown for *P. putida* KT2440. The mutations in *flgL*, *fliA*, *fleQ*, *fliL*, *fliN*, and *flgD* resulted in different degrees of impaired swimming and swarming motility, and affected its adhesion to the corn seeds [44]. Chemotaxis toward exudate components played a major role in the colonization of tomato roots by *P. fluorescens* WCS365 [45]. The *cheA* mutant, defective in flagella-driven chemotaxis, colonized the plant roots less efficiently than the wild-type strain. In turn, type IV pili, which are required for twitching motility, were shown to play a significant role in the colonization of a wide range of plants [44].

The other important characteristic of *P. fluorescens* BRZ63 that underlines its potential for colonization and hence biocontrol of plants is the ability to autoaggregate and form biofilm. Strains of *P. fluorescens* have been reported to coat plant roots by forming a biofilm, which may protect roots against soil bacterial and fungal pathogens [46]. Furthermore, in the root system, bacteria that are found in the biofilm state have several advantages, allowing the organisms that compete in root colonization to create for themselves a better protected niche [47]. Hernández-Salmerón et al. [48] reported that biofilm formation by the *P. fluorescens* UM270 strain played an essential role in root attachment and rhizosphere colonization of *Medicago* spp. plants. To mediate the adhesion and colonization of plant roots, a variety of plant-associated bacteria produce EPS and/or LPS. Meneses et al. [49] reported that the *gumD* gene in endophytic diazotrophic bacterium *G. diazotrophicus* PAL5 was essential for EPS production and biofilm formation. EPS can also contribute to the survival of bacteria within the plant by acting as a barrier against plant defense mechanisms [50]. It is worth noting that the ability to produce the EPS was also observed in the BRZ63 strain. These results were consistent with the identification of a gene cluster involved in alginate biosynthesis in the BRZ63 genome. Additionally, key genes of LPS biosynthesis were detected. According to Chang et al. [51], pseudomonads produce alginate in response to water-limiting conditions, which influences biofilm development and the EPS physiochemical properties. It may facilitate the maintenance of a hydrated microenvironment and protect residents from drought stress, increasing their survival [52]. In turn, structural changes of LPSs have been shown to usually affect adhesive forces among bacteria, possibly through alteration of the cell surface hydrophobicity [53]. The presence of genes coding for enzymes catalyzing degradation of plant cell well polysaccharides and the arsenal of genes encoding key antioxidant enzymes additionally underlines the high colonization potential of the BRZ63 strain and gives it an advantage for survival in a highly oxidative environment. Successful visualization of the EGFP-labeled BRZ63 strain in the inner tissues of *Arabidopsis thaliana* Col-0 confirms its effective colonization properties. The BRZ63 strain extensively colonized the root surface, forming aggregates and biofilm. It was also able to colonize the interior of epidermal cells of the plant.

Once the effective colonization by *P. fluorescens* BRZ63 was demonstrated, we aimed to assess whether this bacterium is able to produce metabolites involved in biocontrol and plant growth promotion. The ability of the BRZ63 strain to produce siderophores and the presence of a number of genes for biosynthesis of bacterioferritin, enterobactin, and pyoverdine as well as iron transport is an extremely important feature that can contribute to the biocontrol effect of the strain. It is known that *Pseudomonas* spp. producing siderophores not only promote plant growth but also contribute to disease control by competing with phytopathogens for trace metals. For example, siderophores produced by *Pseudomonas* spp. and other rhizobacterial organisms (*Bacillus*, *Enterobacter*) have been used in the biological control of damping-off of cotton caused by *Pythium ultimum* [54]. Moreover, results obtained by Zhao et al. [55] suggested a significant positive correlation between siderophore production by endophytic strains of *Enterobacter*, *Acinetobacter*, *Pseudomonas*, and *Bacillus* genera and the inhibition ratio against *Phytophthora sojae* 01. Siderophores are also known to induce systemic resistance in plants. For example, the purified pyoverdine from *P. fluorescens* WCS374 induced resistance in radish against *Fusarium* wilt [56].

Production of lytic enzymes, chitinase, and β-1,3-glucanase as well as biosurfactants of high emulsification activity and great potential for lowering the surface tension are other features that may play a crucial role in the biological control of plant pathogens by the BRZ63 strain. It is speculated that cell wall lysis of the pathogenic fungi is a result of coordinated action of a complex of hydrolytic enzymes. Production of chitinase, β-1,3-glucanases, and protease by four *Bacillus* strains was an important mechanism responsible for *Rizoctonia solani* inhibition in tomato plants [57]. Similarly, the *Bacillus subtilis* strain 330-2 isolated from a rapeseed, which produced β-1,3-glucanase, β-1,4-glucanase, and proteases, strongly suppressed the in vitro growth of *Rhizoctonia solani* AG1-IA, *Botrytis cinerea*, *Fusarium oxysporum*, *Alternaria alternata*, *Cochliobolus heterostrophus*, and *Nigrospora oryzae* [58]. In turn, Goswami et al. [59] showed that rhamnolipid biosurfactant produced by *P. aeruginosa* DS9 has strong antifungal activity against *C. falcatum*, which may offer the possibility of its application as an alternative fungicide to control red rot disease of sugarcane. Moreover, biosurfactants can acts on the lipids in the cell and alter the membrane fluidity. The rhamnolipid biosurfactants produced by *Pseudomonas* strains inhibited the growth of *E. coli*, *B. subtilis*, *S. aureus*, and *S. epidermidis* [60]. The authors suggested that high emulsification potentials might enhance rhamnolipids to penetrate the cell wall of bacteria. In another studies, Abalos et al. [61] tested the antifungal activity of rhamnolipids produced by the *Pseudomonas* AT10 strain. The highest inhibition activity against *Aspergillus niger*, *Botrytis cinerea*, *Colletotrichum gloesporioides*, *Fusarium solani*, *Gliocadium virens*, *Rhizotecnia solani*, and two *Penicillium* strains was observed for surfactants, effectively lowering the surface tension.

The biocontrol effect of the BRZ63 strain could be also exerted by the induction of a systemic resistance response in the host plant since the genome analysis has revealed that the strain has genes required for the biosynthesis of 2,3-butanediol and acetoin together with genes engaged in the biosynthesis of SA through the chorismate/isochorismate pathway. The SA synthesis was linked to the ability of *P. aeruginosa* 7NSK2 to elicit induced systemic resistance (ISR) against *Botrytis cinerea* in bean [62]. It is worth emphasizing that the production of SA by the BRZ63 strain was confirmed by biochemical studies and the strain showed an above 30-fold more effective production of SA than the well-known biocontrol agent PICF7 [63].

*P. fluorescens* BRZ63 was also able to yield positive results in a test for IAA and ammonia production, ACC deaminase synthesis, and phosphate solubilization. IAA is involved in cell–cell signaling, regulation of plant development, and induction of plant defense systems [64]. Etesami et al. [65] reported that bacteria with the ability to produce IAA not only improve plant growth but also seem to colonize plant roots more efficiently. In another study, Hernández-León et al. [66] showed that the strain *P. fluorescens* UM16 producing IAA at a concentration of 22 μg/mL was able to induce shoot growth in *M. truncatula* plants even in the presence of *Botrytis cinerea*. Interestingly, the BRZ63 strain produced higher levels of IAA (59.62 ± 1.11 μg/mL) than *P. fluorescens* UM16, which emphasizes its potential role in plant growth promotion. Based on the genome analysis, we speculate that in the studied strain, IAA synthesis proceeds through the indole-3-acetonitrile (IAN) pathway. The ability of BRZ63 of phosphate solubilization and high PSI are also remarkable features of the strain. The presence of genes encoding the ABC transport complex (PstABCS), which is responsible for inorganic phosphate uptake under phosphate starvation conditions [67], suggests a strong capability of the strain for phosphate uptake from the environment. Furthermore, the genomic analysis enabled identification of the *gcd* gene encoding glucose dehydrogenase and *pqqABCDE* operon for pyrroloquinoline quinone involved in gluconic acid (GA) biosynthesis and its release into the environment. This finding suggests that similarly to three endophytic *Pseudomonas* strains L111, L228, and L321 isolated from *Miscanthus giganteus* [68], production of GA constitutes a major mechanism of phosphate solubilization by the BRZ63 strain.

## 4. Materials and Methods

### 4.1. Bacterial Strains and Growth Conditions

*Pseudomonas fluorescens* BRZ63 was isolated from surface-sterilized roots of the oilseed rape (*Brassica napus* L.) growing in the area contaminated with pesticides in the vicinity of Bielsko-Biała, Southern Poland [49.847665 N, 18.830396 E]. Herein, a laboratory endophytic strain *Enterobacter asburiae* 4FJK isolated from the high hawk (*Hieracium piloselloides*) [69] and *Pseudomonas* sp. P-1 isolated from soil contaminated with petroleum hydrocarbons [70] were used as controls in assays comprising the characteristic of biochemical and physiological features of the BRZ63 strain. Both control strains were derived from the Microbial Culture Collection of the Institute of Biology, Biotechnology and Environmental Protection (Faculty of Natural Sciences, University of Silesia in Katowice, Poland). Bacterial strains used in this study were cultivated routinely on LB Agar or in Luria-Bertani broth (LB Broth) at 30 °C with shaking (130 rpm). For antagonistic activity tests, the BRZ63 strain was grown on potato dextrose agar (PDA) (A&A Biotechnology, Gdynia, Poland).

### 4.2. Isolation and Molecular Identification of the BRZ63 Strain

The BRZ63 strain was isolated from the surface-sterilized roots of the oilseed rape according to the standard protocol [69] with minor modifications. Briefly, the roots of the oilseed rape were washed separately under tap water to remove adhering soil particles, surface sterilized with 70% ethanol (1 min) and 5% sodium hypochlorite (ACE) (5 min), and rinsed three times in sterile distilled water (3 × 5 min). A sterility check was performed by plating 100 µL of sterile water from the final rinse on LB agar medium. For the isolation of bacteria, 2 g of sterilized macerated roots were put into 9 mL of saline solution (0.9% NaCl) and a 100-μL suspension was plated on LB agar medium and incubated at 30 °C for 48 h. After incubation, individual bacterial colonies were isolated and purified. The selected BRZ63 isolate was identified by sequencing of the 16S rRNA gene. The BRZ63 strain was grown in LB medium for 24 h at 30 °C and genomic DNA was extracted using the GeneMatrix Bacterial and Yeast Genomic Purification Kit (EURx, Gdansk, Poland) according to the protocol. Amplification of the 16S rRNA gene was performed with 8F (5′-AGAGTTTGATCCTGGCTCAG-3′) and 1492R (5′-GGTTACCTTGTTACGACTT-3′) universal primers. The PCR master (50 μL) contained: 50 ng of genomic DNA, 1 U of Taq DNA polymerase, 1× TaqDNA polymerase buffer, 0.2 mM of dNTP, 2 mM MgCl_2_, 0.2 μM of the each forward and reverse primers. The PCR cycling conditions were 5 min at 94 °C; 30 cycles of 1 min at 94 °C, 45 s at 54 °C, and 90 s at 72 °C; and 10 min at 72 °C. The PCR-amplified 16S rRNA region was sequenced directly by the commercial company GENOMED (Warsaw, Poland). Obtained sequences were compared with the public databases using NCBI BLASTN online (http://www.ncbi.nlm.nih.gov/). The taxonomic affiliation of the BRZ63 strain was also supported by the genomic sequencing described in Section 4.9 and Section 4.10.

### 4.3. Pathogenic Fungi

*Rhizoctonia solani* W70, *Colletotrichum dematium* K, *Sclerotinia sclerotiorum* K2291, and *Fusarium avenaceum* were derived from the Microbial Culture Collection of the Institute of Biology, Biotechnology and Environmental Protection (Faculty of Natural Sciences, University of Silesia in Katowice, Katowice, Poland). Fungal pathogens were originally isolated from plants tissues exhibiting clear symptoms of diseases. *Rhizoctonia solani W70* was isolated from the grapevine (*Vitis vinifera* L.), *Colletotrichum dematium K* and *Sclerotinia sclerotiorum K2291* were isolated from the caraway (*Carum carvi* L.), and *Fusarium avenaceum* was isolated from wheat (*Triticum* L.). All pathogens were incubated on potato dextrose agar (PDA) (A&A Biotechnology, Gdynia, Poland) at 30 °C.

### 4.4. Antifungal Activity Assays

The antagonistic activity of the endophytic BRZ63 strain was tested by the standard in vitro dual culture assays on PDA medium [71]. *Rhizoctonia solani* W70, *Colletotrichum dematium* K, *Sclerotinia sclerotiorum* K2291, and *Fusarium avenaceum* were grown separately on PDA medium at 30 °C for 5–21 days (depending of the fungal strain). Then, a 5-mm agar-mycelium disk was taken from each actively growing fungal colony and placed 10 mm from the edge of a Petri dish with the PDA, separately for each pathogen. Next, a sterile loop of the overnight culture of the BRZ63 bacterial suspension was taken and streaked 30 mm away from the disc of the fungal pathogen. Petri dishes inoculated only with fungi were used as controls. Control plates and those with bacteria and fungi were incubated for 5–21 days (depending on the fungal strain) at 30 °C. After incubation, the distance between the point of inoculation of the fungal disk and actively growing edges of the fungus was measured in both, control plates and those coinoculated with bacteria. The percent growth inhibition (PGI) was calculated using the equation:

(1) $$\mathrm{PGI}=\frac{\mathrm{KR}-R1}{\mathrm{KR}}\;\times \;100\%,$$

where KR represents the distance (in mm) from the point of the fungal inoculation to mycelium growing edges on control dishes, and R1 represents the distance of fungal growth towards the antagonist from the point of fungal inoculation to the fungal colony margin on plates inoculated with bacteria. The experiment was performed in triplicate.

### 4.5. In Vitro Assessment of Colonization, Plant Growth Promotion (PGP), and Biocontrol Traits of the BRZ63 Strain

*P. fluorescens* BRZ63 was evaluated for its colonization and plant growth-promoting capability as well as biocontrol properties in comparison to the control 4FJK strain. Microbial production of cell wall-degrading enzymes, such as proteases and cellulases, was determined according to the protocol of Vijayalakshmi et al. [72]. Chitinase production was tested on the colloidal chitin agar medium using the method described by Kuddus and Ahmad [73]. The biofilm formation, motility, oxidase, and catalase activity of the tested strains were determined following the procedures described by Naveed et al. [74]. Classification of the biofilm formation ability was performed according to Stepanovic et al. [22]. Production of EPS was assessed by cultivating the BRZ63 and 4FJK on CRA using the method modified by Freeman et al. [75]. Production of indole-3-acetic acid by the tested strains was determined by a colorimetric method using Salkowski reagent [76], while the production of ammonia was determined after the incubation of the tested bacteria in peptone water for 72 h at 30 °C [77]. Production of 1-aminocyclopropane-1-carboxylate deaminase was determined according to the method of Sandhya et al. [33]. Solubilization of phosphate was detected based on the formation of the clear halo surrounding the bacterial colony on the Pikovskay’a medium containing insoluble Ca_3_(PO_4_)_2_ after 5 days of incubation at 30 °C [78] and PSI was calculated according to the method described by [79]. The ability of strains to produce siderophores was determined in CAS agar [80]. Production of acetoin and 2,3-butanediol was determined according to the method of Johnston-Monje and Raizada [81]. The ability of strains to produce SA and hydrogen cyanide (HCN) was verified according to the method of Syamala and Sivaji [82] and Ahmad et al. [83], respectively. The ability of the BRZ63 strain to synthesize extracellular glycolipid biosurfactants and emulsification activity was evaluated in comparison to the control P-1 strain using the method described by Pacwa-Płociniczak et al. [70] and Shoeb et al. [84], respectively. All experiments were performed in three biological replicates.

### 4.6. Autoaggregation Assay

Briefly, the BRZ63 and 4FJK strains were cultivated separately in the LB medium for 24 h at 30 °C and centrifuged (5000 rpm, 20 min, 4 °C). The cells were washed three times to remove the remaining culture medium, resuspended in phosphate-buffered saline (PBS pH 7.2), and the bacterial cell suspension was adjusted to OD at a wavelength of 600 nm (OD_600_) of 1.0. Then, OD_600_ was measured after 2, 18, and 24 h using a spectrophotometer. Aggregation was expressed as a percentage of aggregated cells and calculated as follows:

(2) $$A=\frac{{\mathrm{OD}}_{600i}-{\mathrm{OD}}_{600a}}{{\mathrm{OD}}_{600i}}\;\times \;100\%,$$

where OD_600i_ is the initial absorbance of the bacterial cell suspension and OD_600a_ is the absorbance measured after the incubation.

### 4.7. Plant Colonization Experiments

#### 4.7.1. Fluorescent Labeling of the BRZ63 Strain

To label the BRZ63 strain with the EGFP protein, electrocompetent cells were prepared according to Nigris et al. [85] and transformed with pMP4566 plasmid vector with the constitutive expression of the *egfp* gene [23]. Electrotransformation was performed following the protocol of Prieto and Mercado-Blanco, [86]. Bacteria harboring plasmid were examined using a Nikon ECLIPSE-Ni-U stereo microscope equipped with epifluorescence detection, with 480/40 nm excitation and 510 nm long pass emission filter.

#### 4.7.2. Inoculation of *Arabidopsis thaliana* Col-0 with the EGFP-Labeled Strain

The ability of the tested BRZ63 strain labeled with the EGFP to colonize plants was studied by inoculating seeds of a model plant *Arabidopsis thaliana* Col-0 using the method described by Naveed et al. [74] with minor modifications. The seeds were incubated for 2 h in distilled water and then surface sterilized for 5 min in a 20% sodium hypochlorite solution. Next, the seeds were washed three times in distilled water and then incubated for 2 h in a bacterial cell suspension (OD_600_ = 0.5). Bacteria used for inoculation were grown for 24 h at 30 °C in the LB medium supplemented with tetracycline (at a concentration of 50 μg/mL), subsequently centrifuged (5000 rpm, 10 min), and then suspended in sterile distilled water. Inoculated seeds were plated on water agar plates (1%) and grown for 7 days at room temperature under natural sunlight. In order to confirm the colonization of plants by the tested strain, 6 seedlings were selected. Root explants were first surface sterilized for 2 min with sodium hypochlorite 5%, rinsed with 70% ethanol, and then washed 3 times for 10 min with sterile deionized water. Roots were sliced longitudinally with a blade, mounted on a slide, and covered with a coverslip. The bacterial cells within the plant tissues were observed in a Nikon ECLIPSE-Ni-U stereo microscope equipped with epifluorescence detection, with 480/40 nm excitation and 510 nm long pass emission filter. As a control, the uninoculated plants were analyzed.

### 4.8. Effects of the BRZ63 Strain Inoculation on Oilseed Rape Development and Disease Protection

*B. napus* seeds were surface sterilized and inoculated with the EGFP–labeled BRZ63 strain as described above (Section 4.7.2). Next, bacteria-inoculated and uninoculated sterilized seeds were soaked for 5 h in homogenized mycelial mass separately for each fungal pathogen. The phytopathogens used for preparation of fungal inoculum were grown separately on PDA medium at 30 °C for 5–21 days (depending of the fungal strain). After incubation, the mycelial mass was collected, homogenized (2000 rpm, 2 min on ice) using an IKA Ultra-Turrax T25 Digital Homogenizer, and agitated with 15 mL of sterile distilled water. The prepared seeds were divided into 8 sets of treatments, namely RZ+BRZ63+CD (seeds inoculated with the BRZ63 strain and dipped in the inoculum of *C. dematium* K), RZ+BRZ63+RS (seeds inoculated with the BRZ63 strain and dipped in the inoculum of *R. solani* W70), RZ+BRZ63+SS (seeds inoculated with the BRZ63 strain and dipped in the inoculum of *S. sclerotiorium* K2291), RZ+BRZ63+FA (seeds inoculated with strain BRZ63 and dipped in the inoculum of *F. avenaceum*), CD (uninoculated seeds dipped in the inoculum of *C. dematium K*), RS (uninoculated seeds dipped in the inoculum of *R. solani* W), SS (uninoculated seeds dipped in the inoculum of *S. sclerotiorum* K2291), and FA (uninoculated seeds dipped in the inoculum of *F. avenaceum*), plated on water agar plates (1%) and grown at room temperature under natural sunlight. As positive controls, uninoculated seeds (RZ) and seeds inoculated with the BRZ63 strain (RZ+BRZ63) were used. The germinating seeds and seedlings were observed continuously to identify any phenotypic differences between treatments. The characteristics that were measured included the germination efficiency and the length and weights of the roots and shoots. All experiments were performed using 25 seeds for each treatment, repeated twice.

### 4.9. Genome Sequencing and Sequence Analysis

Genomic DNA of *P. fluorescens* BRZ63 was extracted using GeneMatrix Bacterial and Yeast Genomic Purification Kit (EURx, Gdansk, Poland). The sequencing was performed by MicrobesNG on the Illumina MiSeq platform with 2 × 250-bp paired-end reads. The results of the sequencing were subjected to a standard MicrobesNG analysis pipeline and were deposited in the GenBank database under the accession number SPVI00000000.1. Functional annotation was performed using eggNOG 5.0 with a one-to-one orthology restriction (http:/eggnogdb.embl.de) [87]. For the annotation of gene function, genes were compared to the KEGG (Functional Kyoto Encyclopedia of Genes and Genomes) database [88]. WebMGA was used for functional analysis of Cluster of Orthologous Genes (COG) [89]. antiSMASH 5.0 was used for identification of gene clusters responsible for biosynthesis of secondary metabolites [90]. The CAZy database (Carbohydrate Active Enzymes database, http://www.cazy.org/) was used to classify cell wall-degrading enzymes (CWDEs) and divided them into different families. CAZy families were identified with dbCAN2 according to the DIAMOND database [91,92]. Sequencing data and assembly were submitted to a public NCBI database under the BioProject accession number PRJNA529642.

### 4.10. Phylogenetic Analysis

The phylogenetic tree was constructed based on the type strains and well-described strains belonging to the *Pseudomonas* genus with *Escherichia coli* K-12 substr. MG1655 as an outgroup. The core proteomes of the 20 strains were extracted and aligned with M1CR0B1AL1Z3R on default settings [93]. Poorly aligned regions were removed by Gblocks (version 0.91b) (Integrated DNA Technologies, Iowa, IA, USA) [94], which resulted in 6824 amino acids for 20 genomes. A maximum-likelihood phylogenetic tree was obtained with MEGA X (version 10.1.5) (Pennsylvania State University, Mueller Laboratory, University Park, PA, USA) on default settings and 1000 bootstraps resampling value [95].

### 4.11. Statistical Analysis

The statistical analysis was carried out by using Microsoft Office Excel 2010 and Statistica 12.5 PL (StatSoft^®^ Inc., Tulsa, OK, USA). Data were presented as the mean ± standard deviation (SD) of three biological replicates. Data were analyzed using one-way analysis of variance (ANOVA). Whenever significant differences were observed, a post hoc Tukey’s honest significant difference (HSD) test was used to further elucidate differences among the means (*p* < 0.05). Letters indicate that means differ significantly.

## 5. Conclusions

The results of this study showed that the *P. fluorescens* BRZ63 strain originally isolated from the roots of oilseed rape holds great potential to be exploited as a biological control agent. The examined strain was found to have antagonistic activity against taxonomically diverse fungal pathogens and inhibited the fungal infection in the germinating oilseed rape seeds and seedlings. It can contribute to biocontrol and promotion of the plant growth through a variety of mechanisms, including production of siderophores, lytic enzymes, biosurfactants, SA, IAA, ACC deaminase, ammonia, and phosphate solubilization. Genome sequencing confirmed the presence of crucial genes encoding a wide range of mechanisms determining biological activity, plant growth promotion, and colonization. The potential for the production of a range of secondary metabolites underlines its ability to protect plants against phytopathogens. Antagonistic activity of the BRZ63 towards various pathogens significantly increases the possibility of its application as a biocontrol factor.

## Figures and Tables

**Table 1:** Biochemical and physiological characteristics of endophytic *P. fluorescens* BRZ63.

Features	Strain BRZ63	Strain 4FJK
Exopolysaccharides production	+	−
Siderophore production	+	+
Endoglucanase production Chitinase production Protease production	+	−
	+	−
	−	−
Acetoin and 2,3-butanediol production	−	+
IAA production (μg/mL)	59.62 ± 1.11 ^a^	34.53 ± 0.97 ^b^
SA production (μg/mL)	17.83 ± 0.95 ^a^	55.22 ± 1.36 ^b^
ACC deaminase production	+	+
Ammonia production	+	+
HCN production	−	+
Catalase production	+	−
Oxidase production	+	−

(+) capability, (−) lack of capability. ^a,b^—indicate statistically significant differences (*p* < 0.05) according to the HSD test.

**Table 2:** Qualitative estimation of the phosphate solubilization efficiency of *P. fluorescens* BRZ63 and the control *Enterobacter asburiae* 4FJK strain.

Bacterial Strain	Colony Diameter (mm)	Zone of Solubilization (mm)	Phosphate Solubilization Index (PSI)
BRZ63	7.84 ± 0.2	52.3 ± 0.12 ^a^	7.67 ± 0.33 ^a^
4FJK	10.1 ± 0.3	7.7 ± 0.2 ^b^	1.76 ± 0.19 ^b^

^a,b^—indicate statistically significant differences (*p* < 0.05) according to the HSD test.

**Table 3:** Biosurfactant production by *P. fluorescens* BRZ63 and the control *Pseudomonas* sp. P-1.

Test		Strain BRZ63	Strain P-1
Methylene blue agar test		+	+
Oil-spreading (mm)		40.30 ± 3.50 ^a^	44.01 ± 1.52 ^a^
Emulsification index (%)	diesel oil	32.51 ± 2.53 ^a^	58.33 ± 4.81 ^b^
	cyclohexane	78.79 ± 2.62 ^a^	77.37 ± 2.63 ^a^

(+) the capability of biosurfactant production. ^a,b^—indicate statistically significant differences (*p* < 0.05) according to the HSD test.

**Table 4:** General genome features of *P. fluorescens* BRZ63.

Attribute	Value
Genome size (bp)	6,335,040 bp
Contigs	363
G+C content (%)	64
Genes (total)	6120
CDSs (total)	6043
Genes (coding)	5915
Protein genes	5915
RNA genes	77
rRNAs	7, 3, 1 (5S, 16S, 23S)
Complete rRNAs	7, 1, 1 (5S, 16S, 23S)
Partial rRNAs	2 (16S)
tRNAs	62
ncRNAs	4
Pseudogenes	128
BioProject ID	PRJNA529642
GenBank accession number	SPVI00000000.1

## Supplementary Materials

Supplementary materials can be found at https://www.mdpi.com/1422-0067/21/22/8740/s1. Table S1. Functional cluster of orthologous genes (COG) classification of predicted genes in the *P. fluorescens* BRZ63 strain. Table S2. Secondary metabolite gene clusters identified in the *P. fluorescens* BRZ63 strain using antiSMASH v. 5.1.2. Table S3. Genes attributed to biocontrol, plant growth promotion and colonization traits identified in the P. fluorescens BRZ63 genome. Table S4. Genes related to carbohydrate-active enzymes (CAZymes) in the *P. fluorescens* BRZ63 strain. Table S5. CAZymes involved in plant and fungal cell wall degradation identified in the *P. fluorescens* BRZ63 genome. Figure S1. In vitro inhibition of mycelial growth of *Rhizoctonia solani* W70 (A), *Colletotrichum dematium* K (B), *Sclerotinia sclerotiorum* K2291 (C), and *Fusarium avenaceum* (D) by *P. fluorescens* BRZ63. Upper panel–control - pathogenic fungi grown on PDA medium; bottom panel–dual culture assay on PDA medium - growth of fungi in the presence of BRZ63. Figure S2. Effects of the BRZ63 strain on disease protection of oilseed rape. The leaves and roots of seedlings emerged from the uninoculated seeds (RZ) and seeds inoculated with the BRZ63 strain (RZ+BRZ63). The leaves of seedlings emerged from the uninoculated seeds dipped in the inoculum of *F. avenaceum* (RZ+FA) and seeds preinoculated with strain BRZ63 and dipped in the inoculum of the pathogen (RZ+BRZ63+FA). The leaves of seedlings emerged from the uninoculated seeds dipped in the inoculum of *S. sclerotiorium* K2291 (RZ+SS) and seeds preinoculated with strain BRZ63 and dipped in the inoculum of the pathogen (RZ+BRZ63+SS). The leaves of seedlings emerged from the uninoculated seeds dipped in the inoculum of *C. dematium* K (RZ+CD) and seeds preinoculated with strain BRZ63 and dipped in the inoculum of the pathogen (RZ+BRZ63+CD). The roots of seedlings emerged from the uninoculated seeds dipped in the inoculum of *R. solani* W70 (RZ+RS) and seeds preinoculated with strain BRZ63 and dipped in the inoculum of the pathogen (RZ+BRZ63+RS).

## Abbreviations

IAA	Indole-3-acetic acid
ACC	1-aminocyclopropane-1-carboxylate
HSD	Honest significant difference
CRA	Congo Red Agar
EPS	Exopolysaccharide
CV	Crystal Violet
EGFP	Enhanced Green Fluorescent Protein
SA	Salicylic acid
ISR	Induced Systemic Resistance
DF	Dworkin and Foster
PSI	Phosphate Solubilizing Index
CAS	Chrome azurol S
CTAB	Cetyltrimethylammonium bromide
CDS	Protein-encoding sequences
COG	Cluster of Orthologous Genes
PGP	Plant Growth Promotion
LPS	Lipopolysaccharide
GH	Glycoside Hydrolases
CE	Carbohydrate Esterases
TBDRs	TonB-dependent receptors
GDH	Glutamate dehydrogenase
PQQ	Pyrroloquinoline quinon
LB	Luria-Bertani
PDA	Potato Dextrose Agar
HCN	Hydrogen cyanide
CWDEs	Cell Wall Degrading Enzymes
