# Supplementary material for: Genome Mining and Evaluation of the Biocontrol Potential of Pseudomonas fluorescens BRZ63, a New Endophyte of Oilseed Rape (Brassica napus L.) against Fungal Pathogens
Source: Int J Mol Sci. 2020 Nov 19;21(22):8740. doi: 10.3390/ijms21228740 (PMC7699435; doi:10.3390/ijms21228740)
Supplement: Supplementary file 1 [file ijms-21-08740-s001.pdf]

Table S1

Functional cluster of orthologous genes (COG) classification of predicted genes in the *P. fluorescens* BRZ63 strain

Code	Description	CDS	%CDS
**A**	RNA processing and modification	2	0.03
**B**	Chromatin structure and dynamics	2	0.03
**C**	Energy production and conversion	354	5.95
**D**	Cell cycle control. cell division. chromosome partitioning	54	0.91
**E**	Amino acid transport and metabolism	586	9.85
**F**	Nucleotide transport and metabolism	151	2.54
**G**	Carbohydrate transport and metabolism	324	5.45
**H**	Coenzyme transport and metabolism	213	3.58
**I**	Lipid transport and metabolism	252	4.24
**J**	Translation. ribosomal structure and biogenesis	226	3.80
**K**	Transcription	582	9.79
**L**	Replication. recombination and repair	181	3.04
**M**	Cell wall/membrane/envelope biogenesis	357	6.00
**N**	Cell motility	174	2.93
**O**	Posttranslational modification. protein turnover. chaperones	163	2.74
**P**	Inorganic ion transport and metabolism	451	7.58
**Q**	Secondary metabolites biosynthesis. transport and catabolism	178	2.99
**S**	Function unknown	1090	18.33
**T**	Signal transduction mechanisms	356	5.99
**U**	Intracellular trafficking. secretion. and vesicular transport	177	2.98
**V**	Defense mechanisms	74	1.24
**Z**	Cytoskeleton	2	0.03

Table S2

Secondary metabolite gene clusters identified in the *P. fluorescens* BRZ63 strain using antiSMASH v. 5.1.2

Cluster	Type	From	To	Most similar known cluster	Similarity
Cluster 1	LAP	85.954	107.934		
Cluster 2	NAGGN	330.566	345.306		
Cluster 3	NRPS	373.569	426.477	pyoverdin	9%
Cluster 4	siderophore	512.834	524.762		
Cluster 5	bacteriocin	326.216	337.061		
Cluster 6	arylpolyene	232.497	276.072	APE Vf	45%
Cluster 7	bacteriocin	41.058	51.936		
Cluster 8	NRPS	1	24.586	viscosin	37%
Cluster 9	betalactone	45.049	73.349	fengycin	13%
Cluster 10	terpene	131.565	152.575		
Cluster 11	NRPS	64.606	101.624	taiwachelin	11%
Cluster 12	NRPS-like	51.064	77.559	L-2-amino-4-methoxy-trans-3-butenoic acid	40%
Cluster 13	NRPS	1	7.725		
Cluster 14	NRPS	1	3.435		

Table S3. Genes attributed to biocontrol, plant growth promotion and colonization traits identified in the *P. fluorescens* BRZ63 genome

Gene	Accession number	Gene product	Activity
entD	TFW40873.1	enterobactin synthetase component D [EC:6.3.2.14 2.7.8.-]	Siderophore biosynthesis
iucB	TFW45226.1	acetyl CoA:N6-hydroxylysine acetyl transferase [EC:2.3.1.102]	
bfr	TFW40338.1	bacterioferritin [EC:1.16.3.1]	
bfd	TFW42906.1	bacterioferritin-associated ferredoxin	
fiu	TFW43421.1	catecholate siderophore receptor	Siderophore uptake
exbB	TFW41374.1	biopolymer transport protein ExbB	
exbD	TFW41375.1	biopolymer transport protein ExbD	
tonB	TFW40662.1	periplasmic protein TonB	
pvdA	TFW44939.1	L-ornithine N5-monooxygenase [EC:1.14.13.195 1.14.13.196]	Pyoverdin biosynthesis
pvdE	TFW42495.1	putative pyoverdin transport system ATP-binding/permease protein	
pvdH	TFW45216.1	belongs to the class-III pyridoxal-phosphate-dependent aminotransferase family	
pvdL	TFW45223.1	COG0318 Acyl-CoA synthetases (AMP-forming) AMP-acid ligases II	
pvdG	TFW45224.1	thioesterase	
pvdS	TFW45225.1	belongs to the sigma-70 factor family. ECF subfamily	
pvdJ	TFW42491.1	AA-adenyl-dom amino acid adenylation domain protein	
pvdO	TFW42496.1	Chromophore maturation protein PvdO	
pvdN	TFW42497.1	class V aminotransferase	
pvdM	TFW42498.1	membrane dipeptidase (Peptidase family M19)	
pvdP	TFW42499.1	PvdJ PvdD PvdP-like protein	
pvdI	TFW40457.1	peptide synthetase	
pvdQ	TFW42063.1	protein related to penicillin acylase	
menF	TFW35465.1	isochorismate synthase	Salicylic acid production
pchB	TFW41741.1	isochorismate pyruvate lyase [EC:4.2.99.21]	
afuA	TFW44456.1	iron(III) transport system substrate-binding protein	Iron transport, receptors and related protein
afuB	TFW44457.1	iron(III) transport system permease protein	

afuC	TFW44458.1	iron(III) transport system ATP-binding protein [EC:7.2.2.7]	
-	TFW43370.1	TC.FEV.OM; iron complex outermembrane recepter protein	
-	TFW40302.1	ABC.FEV.S; iron complex transport system substrate-binding protein	
pqqE	TFW42428.1	PqqA peptide cyclase [EC:1.21.98.4]	Phosphate solubilization
pqqD	TFW42429.1	pyrroloquinoline quinone biosynthesis protein D	
pqqC	TFW42430.1	pyrroloquinoline-quinone synthase [EC:1.3.3.11]	
pqqB	TFW42431.1	pyrroloquinoline quinone biosynthesis protein B	
pqqA	TFW42489.1	pyrroloquinoline quinone biosynthesis protein A	
phnA	TFW40776.1	protein PhnA	
ppk2	TFW45161.1	polyphosphate kinase (ADP) [EC:2.7.4.-]	
phoU	TFW44677.1	phosphate transport system protein	
pstB	TFW44678.1	phosphate transport system ATP-binding protein [EC:7.3.2.1]	
pstA	TFW44679.1	phosphate transport system permease protein	
pstC	TFW44680.1	phosphate transport system permease protein	
pstS	TFW44681.1	phosphate transport system substrate-binding protein	
phoQ	TFW40870.1	two-component system. OmpR family. sensor histidine kinase PhoQ [EC:2.7.13.3]	
phoP	TFW40871.1	two-component system. OmpR family. response regulator PhoP	
Pit	TFW42726.1	low-affinity inorganic phosphate transporter	
pqqL	TFW37848.1	zinc protease [EC:3.4.24.-]	
gcd	TFW42967.1	quinoprotein glucose dehydrogenase [EC:1.1.5.2]	
-	TFW41813.1	acetolactate synthase I/II/III large subunit [EC:2.2.1.6]	Production volatile organic compounds
-	TFW41814.1	acetolactate synthase I/III small subunit [EC:2.2.1.6]	
bdh	TFW40243.1	(R.R)-butanediol dehydrogenase / meso-butanediol dehydrogenase / diacetyl reductase [EC:1.1.1.4 1.1.1.- 1.1.1.303]	
acdS	TFW41243.1	1-aminocyclopropane-1-carboxylate deaminase [EC:3.5.99.7]	ACC deaminase activity
-	TFW41148.1	nitrilase [EC:3.5.5.1]	IAA production
oxd	TFW40578.1	aldoxime dehydratase [EC:4.99.1.5	
-	TFW44414.1	oxidoreductase	
trpE	TFW42468.1	anthranilate synthase component I [EC:4.1.3.27]	Antibiotics and secondary metbolites
trpG	TFW42469.1	anthranilate synthase component II [EC:4.1.3.27]	
trpD	TFW42470.1	anthranilate phosphoribosyltransferase [EC:2.4.2.18]	

trpC	TFW42471.1	indole-3-glycerol phosphate synthase [EC:4.1.1.48]	
trpA	TFW44799.1	tryptophan synthase alpha chain [EC:4.2.1.20]	
trpB	TFW44800.1	tryptophan synthase beta chain [EC:4.2.1.20]	
phzF	TFW40232.1	trans-2.3-dihydro-3-hydroxyanthranilate isomerase	
prtB	TFW41941.1	oligopeptidase B [EC:3.4.21.83]	
ubiC	TFW44667.1	chorismate--pyruvate lyase [EC:4.1.3.40]	
ubiA	TFW44668.1	4-hydroxybenzoate polyprenyltransferase [EC:2.5.1.39]	
gapP	TFW43975.1	GABA permease	
pys2	TFW42890.1	pyocin	
-	TFW40521.1	endoglucanase [EC:3.2.1.4]	Lytic enzymes production
bglX	TFW39167.1	beta-glucosidase [EC:3.2.1.21]	
nagAl	TFW43285.1	N-acetylglucosamine-6-phosphate deacetylase (Chitinase activity)	
pvdQ	TFW42063.1	acyl-homoserine-lactone acylase [EC:3.5.1.97]	Quorum quenching
iscU	TFW39818.1	nitrogen fixation protein NifU and related proteins	Nitrogen fixication
manD	TFW41120.1	mannitol 2-dehydrogenase [EC:1.1.1.67]	Mannitol dehydrogenase
rhlB	TFW43588.1	ATP-dependent RNA helicase RhlB [EC:3.6.4.13]	Rhamnolipide production
rhlE	TFW42321.1	ATP-dependent RNA helicase RhlE [EC:3.6.4.13]	
rhlA	TFW42353.1	Alpha beta hydrolase	
algA	TFW43571.1	mannose-1-phosphate guanylyltransferase / mannose-6-phosphate isomerase [EC:2.7.7.13 5.3.1.8]	Exopolisacharydes biosynthesis
algF	TFW43572.1	alginate O-acetyltransferase complex protein AlgF	*Alginate*
algJ	TFW43573.1	alginate O-acetyltransferase complex protein AlgJ	
algI	TFW43574.1	alginate O-acetyltransferase complex protein AlgI	
algL	TFW43575.1	poly(beta-D-mannuronate) lyase [EC:4.2.2.3]	
algX	TFW43576.1	alginate biosynthesis protein AlgX	
algG	TFW43577.1	mannuronan 5-epimerase [EC:5.1.3.37]	
algE	TFW43578.1	alginate production protein	
alg44	TFW43579.1	mannuronan synthase [EC:2.4.1.33]	
alg8	TFW43580.1	mannuronan synthase [EC:2.4.1.33]	
algD	TFW43581.1	GDP-mannose 6-dehydrogenase [EC:1.1.1.132]	
algI	TFW40983.1	alginate O-acetyltransferase complex protein AlgI	

flhG	TFW45253.1	flagellar biosynthesis protein FlhG	Cell Motility
flhF	TFW45254.1	flagellar biosynthesis protein FlhF	*Flagllar assembly*
flhA	TFW45255.1	flagellar biosynthesis protein FlhA	
flhB	TFW45258.1	flagellar biosynthetic protein FlhB	
fliR	TFW45259.1	flagellar biosynthetic protein FliR	
fliQ	TFW45260.1	flagellar biosynthetic protein FliQ	
fliP	TFW45261.1	flagellar biosynthetic protein FliP	
fliOZ	TFW45262.1	flagellar protein FliO/FliZ	
fliNY	TFW45263.1	flagellar motor switch protein FliN/FliY	
fliM	TFW45264.1	flagellar motor switch protein FliM	
fliL	TFW45265.1	flagellar FliL protein	
fliK	TFW45266.1	flagellar hook-length control protein FliK	
fliJ	TFW45270.1	flagellar FliJ protein	
filI	TFW45271.1	flagellum-specific ATP synthase [EC:7.4.2.8]	
fliH	TFW45272.1	flagellar assembly protein FliH	
fliG	TFW45273.1	flagellar motor switch protein FliG	
fliF	TFW45274.1	flagellar M-ring protein FliF	
fliE	TFW45275.1	flagellar hook-basal body complex protein FliE	
fliS	TFW45279.1	flagellar protein FliS	
fliD	TFW45280.1	flagellar hook-associated protein 2	
fliC	TFW45282.1	flagellin	
flrC	TFW45276.1	two-component system. response regulator FlrC	
fleQ	TFW45277.1	sigma-54 dependent transcriptional regulator. flagellar regulatory protein	
fleL	TFW45284.1	flagellar hook-associated protein 3 FlgL	
flgK	TFW45285.1	flagellar hook-associated protein 1 FlgK	
flgJ	TFW45286.1	flagellar protein FlgJ	
flgI	TFW45287.1	flagellar P-ring protein precursor FlgI	
flgH	TFW45288.1	flagellar L-ring protein precursor FlgH	
flgG	TFW45289.1	flagellar basal-body rod protein FlgG	
flgF	TFW45290.1	flagellar basal-body rod protein FlgF	
motB	TFW43788.1	chemotaxis protein MotB	

motA	TFW43789.1	chemotaxis protein MotA	
pilO	TFW43881.1	type IV pilus assembly protein PilO	*Pillus assembly*
pilN	TFW43882.1	type IV pilus assembly protein PilN	
pilM	TFW43883.1	type IV pilus assembly protein PilM	
fimA	TFW43898.1	fimA; major type 1 subunit fimbrin (pilin)	
pilQ	TFW43990.1	type IV pilus assembly protein PilQ	
pilV	TFW43387.1	type IV pilus assembly protein PilV	
pilE	TFW43390.1	type IV pilus assembly protein PilE	
pilD	TFW43404.1	leader peptidase (prepilin peptidase) / N-methyltransferase [EC:3.4.23.43 2.1.1.-]	
pilC	TFW43405.1	type IV pilus assembly protein PilC	
pilA	TFW43652.1	type IV pilus assembly protein PilA	
pilL	TFW44166.1	type IV pili sensor histidine kinase and response regulator	
pilT	TFW42281.1	twitching motility protein PilT	
pilG	TFW42290.1	twitching motility two-component system response regulator PilG	
pilH	TFW42291.1	twitching motility two-component system response regulator PilH	
pilI	TFW42292.1	twitching motility protein PilI	
pilJ	TFW42293.1	twitching motility protein PilJ	
pilL	TFW42294.1	type IV pili sensor histidine kinase and response regulator	
pilF	TFW39810.1	type IV pilus assembly protein PilF	
cheR	TFW42220.1	chemotaxis protein methyltransferase CheR [EC:2.1.1.80]	*Chemotaxis*
cheB	TFW42221.1	two-component system. chemotaxis family. protein-glutamate methylesterase/glutaminase [EC:3.1.1.61 3.5.1.44]	
cheW	TFW45243.1	purine-binding chemotaxis protein CheW	
cheB	TFW45248.1	two-component system. chemotaxis family. protein-glutamate methylesterase/glutaminase [EC:3.1.1.61 3.5.1.44]	
cheA	TFW45249.1	two-component system. chemotaxis family. sensor kinase CheA [EC:2.7.13.3]	
cheZ	TFW45250.1	chemotaxis protein CheZ	
cheY	TFW45251.1	two-component system. chemotaxis family. chemotaxis protein CheY	
cheV	TFW43560.1	cheV; two-component system. chemotaxis family. chemotaxis protein CheV	
aer	TFW45380.1	aer; aerotaxis receptor	
mcp	TFW42247.1	methyl-accepting chemotaxis protein	

lpxL	TFW45209.1	Kdo2-lipid IVA lauroyltransferase/acyltransferase [EC:2.3.1.241 2.3.1.-]	Lypopolysaccharides
lpxO	TFW44377.1	lpxO; beta-hydroxylase [EC:1.14.11.-]	
lpxC	TFW43550.1	lpxC; UDP-3-O-[3-hydroxymyristoyl] N-acetylglucosamine deacetylase [EC:3.5.1.108]	
lpxB	TFW42797.1	lpxB; lipid-A-disaccharide synthase [EC:2.4.1.182]	
lpxA	TFW42798.1	lpxA; UDP-N-acetylglucosamine acyltransferase [EC:2.3.1.129]	
lpxD	TFW42800.1	lpxD; UDP-3-O-[3-hydroxymyristoyl] glucosamine N-acyltransferase [EC:2.3.1.191]	
lpxO	TFW41929.1	lpxO; beta-hydroxylase [EC:1.14.11.-]	
lpxH	TFW41277.1	lpxH; UDP-2.3-diacylglucosamine hydrolase [EC:3.6.1.54]	
oxyR	TFW44636.1	LysR family transcriptional regulator. hydrogen peroxide-inducible genes activator	Oxidative/Stress response
sod2	TFW43483.1	superoxide dismutase. Fe-Mn family [EC:1.15.1.1]	
grxC	TFW43955.1	glutaredoxin 3	
ggt	TFW43320.1	gamma-glutamyltranspeptidase / glutathione hydrolase [EC:2.3.2.2 3.4.19.13]	
soxR	TFW41413.1	MerR family transcriptional regulator. redox-sensitive transcriptional activator SoxR	
gst	TFW45096.1	glutathione S-transferase [EC:2.5.1.18]	
katE	TFW44834.1	catalase [EC:1.11.1.6]	
gpx	TFW45311.1	glutathione peroxidase [EC:1.11.1.9]	
gsr	TFW42192.1	glutathione reductase (NADPH) [EC:1.8.1.7]	
gshB	TFW42289.1	glutathione synthase [EC:6.3.2.3]	

Table S4

Genes related to carbohydrate-active enzymes (CAZymes) in the *P. fluorescens* BRZ63 strain

Familly of enzymes	CBM	GH	GT	PL	AA	CE
Number of genes	14	51	61	2	6	8

CBM. Carbohydrate-Binding Module; GH. Glycoside Hydrolase; GT. Glycosyl Transferase; PL. Polysaccharide lyases; AA. Auxiliary Activity; CE. Carbohydrate Esterase;

Table S5

CAZymes involved in plant and fungal cell wall degradation identified in the *P. fluorescens* BRZ63 genome

Substrate	Family enzymes	Annotation	Copy number
Beta-1-3-glucan	GH17	glucan endo-1.3-β-glucosidase (EC 3.2.1.39)	2
Cellulose	GH3	β-glucosidase (EC 3.2.1.21)	4
Cellulose	GH6	endoglucanase (EC 3.2.1.4)	4
Hemicellulose	GH36	α-galactosidase (EC 3.2.1.22)	1
Pectin	GH28	polygalacturonase (EC 3.2.1.15)	2
Polysaccharides	GH13	α-amylase (EC 3.2.1.1)	11
Polysaccharides	GH15	glucoamylase (EC 3.2.1.3)	1

Figure S1

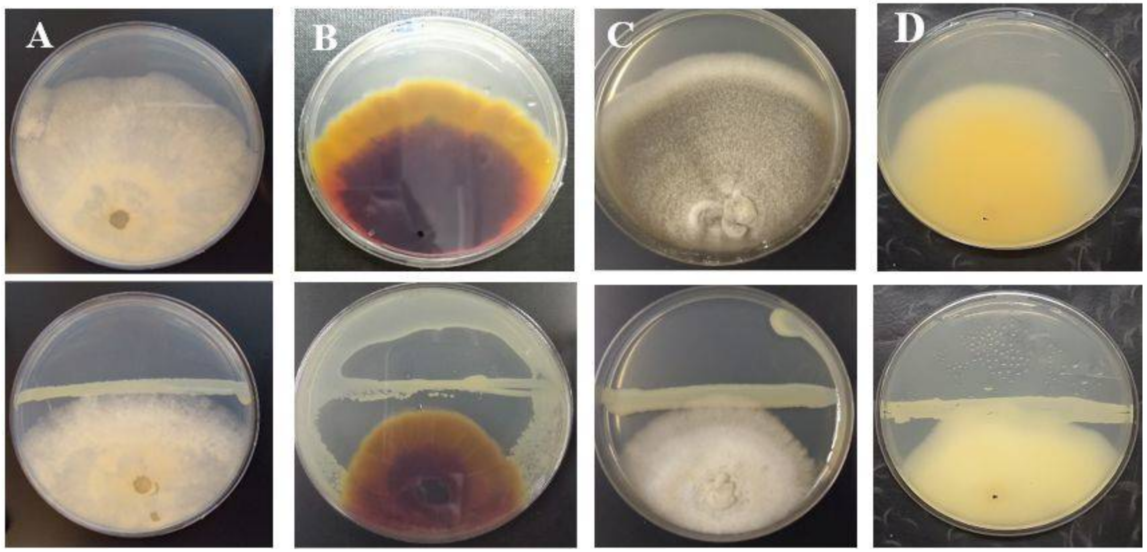

Figure S1. *In vitro* inhibition of mycelial growth of *Rhizoctonia solani* W70 (A), *Colletotrichum dematium* K (B), *Sclerotinia sclerotiorum* K2291 (C), and *Fusarium avenaceum* (D) by *P. fluorescens* BRZ63. Upper panel – control - pathogenic fungi grown on PDA medium; bottom panel – dual culture assay on PDA medium - growth of fungi in the presence of BRZ63.

Figure S2

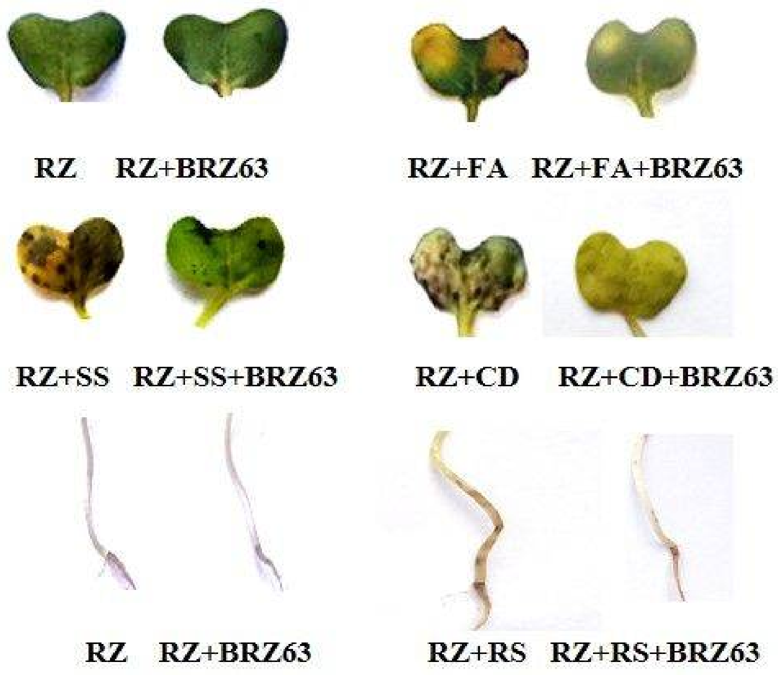

Figure S2. Effects of the BRZ63 strain on disease protection of oilseed rape. The leaves and roots of seedlings emerged from the uninoculated seeds (RZ) and seeds inoculated with the BRZ63 strain (RZ+BRZ63). The leaves of seedlings emerged from the uninoculated seeds dipped in the inoculum of *F. avenaceum* (RZ+FA) and seeds preinoculated with strain BRZ63 and dipped in the inoculum of the pathogen (RZ+BRZ63+FA). The leaves of seedlings emerged from the uninoculated seeds dipped in the inoculum of *S. sclerotiorium* K2291 (RZ+SS) and seeds preinoculated with strain BRZ63 and dipped in the inoculum of the pathogen (RZ+BRZ63+SS). The leaves of seedlings emerged from the uninoculated seeds dipped in the inoculum of *C. dematium* K (RZ+CD) and seeds preinoculated with strain BRZ63 and dipped in the inoculum of the pathogen (RZ+BRZ63+CD). The roots of seedlings emerged from the uninoculated seeds dipped in the inoculum of *R. solani* W70 (RZ+RS) and seeds preinoculated with strain BRZ63 and dipped in the inoculum of the pathogen (RZ+BRZ63+RS).
